# The patient journey to diagnosis and treatment of congenital sucrase-isomaltase deficiency

**DOI:** 10.1007/s11136-021-02819-z

**Published:** 2021-03-27

**Authors:** Heather Smith, Beverly Romero, Emuella Flood, Anne Boney

**Affiliations:** 1 QOL Medical, Raleigh, NC USA; 2 ICON Plc, Gaithersburg, MD USA

**Keywords:** CSID, Sacrosidase, Congenital Sucrase-Isomaltase Deficiency, HRQL, Pediatrics, Adults

## Abstract

**Purpose:**

Congenital sucrase-isomaltase deficiency (CSID) is a rare genetic disorder characterized by a deficiency of the sucrase-isomaltase (SI) enzyme complex within the brush border membrane of the small intestine. Mutations in the *SI* gene result in abnormal synthesis and/or incorrect transport of the SI enzyme. Patients with CSID generally have reduced sucrase activity, but levels of isomaltase activity range from absent to almost normal. This study sought to better understand the experience of patients with CSID prior to, during, and after their diagnosis and its subsequent treatment with sacrosidase.

**Methods:**

This was a cross-sectional interview study conducted in conjunction with a longitudinal, observational study of US patients prescribed and taking sacrosidase for at least three consecutive months as treatment for CSID. The observational study included both children and adults.

**Results:**

This qualitative interview study explored the experiences of 43 adult and pediatric patients (*n* = 8 adults and *n* = 35 children/adolescents) with CSID pre-, during, and post-diagnosis. Findings suggest that a CSID diagnosis is particularly problematic given the disparate range of more commonly understood gastrointestinal (GI) disorders. After diagnosis and treatment with sacrosidase, participants reported considerable improvement in symptoms and health-related quality of life (HRQL), yet symptoms persist that continue to affect daily life, indicating areas of potential unmet need.

**Conclusion:**

Educating clinicians about CSID may help improve the overall diagnosis experience. As this research is the first of its kind in CSID, additional research, qualitative and quantitative, will be important to furthering the understanding of HRQL impact and unmet need experienced by this population and identifying ways to best meet those needs.

## Introduction

Congenital sucrase-isomaltase deficiency (CSID) is a rare genetic disorder characterized by a deficiency of the sucrase-isomaltase (SI) enzyme complex within the brush border membrane of the small intestine [1–3]. Mutations in the *SI* gene result in abnormal synthesis of the SI enzyme and/or incorrect transport of the SI enzyme protein [4, 5]. Patients with CSID generally have reduced sucrase activity, but levels of isomaltase activity range from absent to almost normal [5]. *SI* catalyzes the final step of carbohydrate digestion by breaking disaccharides into absorbable monosaccharides [6].

A deficiency of *SI* can lead to carbohydrate malabsorption characterized by watery, osmotic-fermentative diarrhea, abdominal pain, bloating, flatulence, or cramps [6]. In more severe cases, patients may experience chronic malnutrition and failure to thrive [7]. CSID generally becomes apparent after an infant is weaned and sucrose and starch-containing foods, such as fruits, juices, and grains, are introduced into the diet [8]. However, some patients may experience less severe gastrointestinal (GI) symptoms from birth or for many years before being diagnosed late in childhood or adulthood [9, 10].

The prevalence of CSID is not clear. Historically, estimates have placed the prevalence to be 0.05% to 0.2% in North American and European populations [11, 12], with a higher prevalence (3–10%) in the native populations of Greenland, Alaska, and Canada [8, 13, 14]. However, symptoms can vary from mild to severe and many individuals with the condition remain undiagnosed. Consequently, the actual prevalence of CSID may be much higher than current figures suggest [11, 15].

There are several options for health care providers to aid in their diagnosis of CSID. The standard test for diagnosing CSID is a small bowel biopsy, assayed for disaccharidase activity [11]. Classic CSID results typically show reduced or absent sucrase, reduced isomaltase and palatinase, normal lactase, and normal histology [11]. However, if all four enzymes are low as in the case of pan-disaccharidase deficiency, CSID should not be ruled out. Twenty five percent of the subjects in this observational study reported they had lactose intolerance. Therefore, two conditions, CSID and lactose intolerance, may co-exist [16].

The biopsy technique is invasive and other less invasive tests include the sucrose hydrogen methane breath test or the ^13^C-sucrose breath test, where a measured amount of a sucrose substrate is ingested. The amount of expired hydrogen, methane, or ^13^CO_2_ is measured, respectively [11, 17, 18]. Another approach is to sequence exons of the *SI* gene, which can identify homozygous and compound heterozygote mutations that have been well characterized [5, 19]. A positive genetic test for those known mutations supports the diagnosis of CSID. There is limited *SI* genetic research and therefore, it is possible that every mutation has not been identified to date. A negative test is not conclusive for absence of the disorder. Lastly, a low-sucrose, low-starch diet or an oral enzyme replacement trial using exogenous sucrase (sacrosidase) has also been used to determine if the symptoms of SI deficiency are alleviated by these measures.

The management of CSID is challenging. Treatment with sucrose- and starch-restricted diets can be particularly difficult due to the high sucrose and starch content of the typical Western diet, the lack of information related to the type of sugar and starch content in food, and the lack of access to nutritional education [11, 20]. Enzyme supplementation with sacrosidase has been used to relieve clinical symptoms and sucrose malabsorption in CSID patients [11, 21–23]. Currently, sacrosidase oral solution is the only FDA-approved enzyme replacement therapy for those with genetically determined sucrase deficiency [11, 21–23]. However, sacrosidase does not replace endogenous isomaltase, and patients report lingering symptoms, particularly after ingesting starch [11].

To date, there appears to be no published qualitative research that has investigated the specific challenges faced by patients with CSID or their caregivers. However, research conducted with patients who have other gastrointestinal conditions (e.g., celiac disease) that also require strict dietary restrictions for symptom management indicates that impacts on patients’ daily life and overall quality of life can be high, affecting all areas of life, including relationships, social activities, and emotional functioning [24–26].

While it was expected that patients who managed CSID through diet and sacrosidase would experience improvements in symptoms and overall health-related quality of life, the extent to which these improvements were experienced was unknown. Patient-centered research is increasingly becoming important among the medical community [27, 28]. Qualitative research with patients/caregivers is particularly important in the study of rare diseases, such as CSID, as natural history data are often lacking [29]. Given the lack of knowledge on CSID from the patient perspective, this study sought to fill the gap in natural history data and better understand the experience of patients with CSID prior to, during, and after their diagnosis of this disorder and its subsequent treatment with sacrosidase.

## Methods

This was a cross-sectional interview study conducted in conjunction with a longitudinal, observational study of US patients prescribed and taking sacrosidase for at least three consecutive months as treatment for CSID. The observational study included both children and adults [30]. The study received IRB approval from Salus IRB (Austin, TX).

### Participant selection

All adult patients (18 years and older) and caregivers of children through age 17 years who were enrolled in the study participated in the interviews. While caregivers served as the primary reporters for pediatric patients, all pediatric patients aged 8 – 17 years were also invited to be interviewed, if willing. The target sample was a total of 50 patients. In addition to needing to be located in the US and to being treated with sacrosidase, in order to participate, patients and caregivers had to be able to read, speak, and verbally understand English, have daily access to the Internet and a functional email address, as well as access to an Apple or Android smartphone or tablet.

### Study procedures

All study participants were recruited, screened, consented, and scheduled for a 60–90-min interview between Day 8 and 13 of the observational study. All participants were assigned a unique study ID number to ensure anonymity. All interviews were conducted by trained and experienced qualitative researchers and took place between November 2014 and August 2015.

Interviewers followed semi-structured interview guides designed specifically for each of the following subgroups: adult patients, adult caregivers of pediatric patients, and pediatric patients. Interviews began with open-ended questions to elicit spontaneous feedback, followed by more specific probes. The interviewer elicited information regarding the participants’ experiences with healthcare providers (HCPs), diagnostics, treatment (sacrosidase and diet), gastrointestinal (GI) symptoms, and their health-related quality of life (HRQL).

### Analysis

Verbatim transcripts of the audio-recordings were developed to perform the analysis. A content and thematic analysis was conducted to evaluate the information gathered during the interviews [31]. The analysis consisted of reading the transcript data and applying codes to identify and categorize concepts and themes emerging from the data using MAXQDA [32], a software tool for qualitative data analysis.

In preparation for the qualitative data analysis, a member of the project team read the transcripts and listened to the audio of the interviews to ensure the transcripts accurately reflected the interview conversation. A draft codebook was developed by a lead coder based on the interview guide. The lead coder trained two study team members on the codebook and provided code definitions and examples of how to apply the codes. The two coders then independently coded the first transcript and met periodically with the lead coder and each other to further refine the codebook and ensure inter-coder agreement and understanding of codes. Once the codebook was refined, the remaining transcripts were coded once by the trained coders. New codes were added to the codebook as needed during the coding process, as new concepts emerged, and any coding issues were communicated to the lead coder for resolution. Concepts and themes emerging from the analysis were recorded in a grid to evaluate saturation (i.e., the point at which no new relevant concepts are identified), which is the standard approach used in qualitative research to support the adequacy of the sample size.

Descriptive statistics were used to summarize the sociodemographic and clinical characteristics, which were obtained during screening and enrollment in the observational study.

## Results

### Participant characteristics

A total of 50 interviews representing 43 patients with CSID were conducted. This sample included eight adult patients and 35 caregivers of children/adolescents with CSID, seven of which were dyad interviews that also included a separate interview with the child/adolescent patient. The mean age of all pediatric patients represented was 6.78 years (4.52 STD) and the mean age of adult participants was 28.38 years (10.83 STD); 62.8% of all patients were male. Participants were distributed across the US. Patient sociodemographic characteristics are provided in Table 1.

**Table 1:** Sociodemographic Characteristics

	*All Pediatric patients (*n* = 35)	**Pediatric Interview Participants (*n* = 7)	Adult Patients (*n* = 8)	All Patients (*N* = 43)
Age (years)				
Mean (SD)	6.75 (4.52)	13.14 (2.34)	28.38 (10.83)	10.78 (10.42)
Median	5	13	23	7
Range	1–16	10–16	19–45	1–45
Gender				
Males	26 (74.3%)	6 (85.7%)	1 (12.5%)	27 (62.8%)
Females	9 (25.7%)	1 (14.3%)	7 (87.5%)	16 (37.2%)
Time Zone				
Eastern	15 (42.9%)	3 (42.9%)	5 (62.5%)	20 (46.5%)
Central	11 (31.4%)	2 (28.6%)	1 (12.5%)	12 (27.9%)
Mountain	6 (17.1%)	1 (14.3%)	1 (12.5%)	7 (16.3%)
Pacific	3 (8.6%)	1 (14.3%)	1 (12.5%)	4 (9.3%)

*All pediatric patients includes those children who are represented only by caregivers and those who participated in interviews themselves

**Includes only the subset of pediatric participants who participated in the interviews themselves

Information saturation refers to the point in the interview process when interviews are no longer yielding new information. Out of a total of 34 symptoms (full sample), over 90% (31/34, 91.2%) were identified by the 25th interview, 97.1% (33/34) identified by the end of the 30th interview, and 100% of signs/symptoms reported by the end of the 31st interview.

### Pre-diagnosis and diagnostic period

Participants reported a range of signs/symptoms leading to a CSID diagnosis. For both adults and pediatric patients, the most commonly reported sign/symptom leading to diagnosis was diarrhea (*n* = 6, 75% adults; *n* = 22, 63% children; *n* = 28, 65% total sample). Lack of weight gain was the second most frequently reported symptom leading to diagnosis reported for pediatric patients (*n* = 13, 37%) and the third most common for adults (*n* = 3, 38%), while abdominal pain was the second most reported for adults (*n* = 4, 50%) and fifth for pediatric patients (*n* = 6, 17%). Other key symptoms leading to diagnosis included: reflux (*n* = 1, 13% adults; *n* = 11, 31% for children); growth delays (*n* = 8, 23% children-only), and bloating (*n* = 1, 13% adults; *n* = 7, 20% children). Ten participants (23%) did not discuss symptoms leading to diagnosis.

The majority of participants, including the adult sample, received their CSID diagnosis in childhood or adolescence, though the exact age varied. Two adults (25%) reported receiving their diagnosis in adulthood. Most patients had been diagnosed before the age of five (*n* = 25, 71% children; *n* = 5, 63% adults).

About 39% (*n* = 17) of patients (*n* = 4, 50% adults; *n* = 13, 37% children) received a CSID diagnosis within one year, and 30% (*n* = 13) of participants (*n* = 1, 13% adults; n = 12, 34% children) took between one and two years. For several participants (*n* = 3, 38%; *n* = 3, 9% children; *n* = 6, 14% total sample), receiving the correct diagnosis took five years or more. Participants reported a variety of causes for a lengthy diagnosis process, including practitioners’ lack of knowledge or experience with CSID and signs and symptoms that were mistaken for other medical diagnoses.

- *…so much work chasing what you kind of knew from the beginning…it was very sad and very angering because…it shouldn’t be this hard, and it shouldn’t have to kind of work this hard to get doctors to pay attention to a child… Just because you [the doctor] don’t know it or understand it or haven’t seen it before, shouldn’t be like, you don’t have it …(Caregiver)*

Participants reported seeing a range of health care providers (HCPs) during the diagnostic process. Gastroenterologists (*n* = 30; 70%), pediatricians (*n* = 24; 56%), and general practitioners (*n* = 9, 21%), comprised the majority of health care provider types.

The challenge in securing a diagnosis was also represented by the number of HCPs that participants visited prior to obtaining a CSID diagnosis. Almost a third of participants (*n* = 2, 25% adults; *n* = 11, 31% children; *n* = 13, 30% total sample) reported visiting four or more doctors during the diagnostic process. Patients were subjected to a variety of tests that sought to eliminate a list of more commonly understood health issues, which most commonly included biopsy, endoscopy and colonoscopy, as well as blood, stool, breath, allergy, and genetic testing. Before receiving the ultimate diagnosis of CSID, many other conditions were ruled out, the most frequently of which was celiac disease (*n* = 2, 25%; adults; *n* = 15, 43% children; *n* = 17, 40% total sample), followed by lactose intolerance (*n* = 1, 13% adults; *n* = 12, 34% children; *n* = 13; 30%). For adults specifically, irritable bowel syndrome (IBS) was ruled out most frequently (*n* = 5, 63% adults; *n* = 2, 6% children).

### Post-diagnosis and treatment period

After diagnosis, participants generally described treatment for CSID as a combination of taking sacrosidase and diet modifications of sucrose and starch-containing foods.

Treatment with sacrosidase improved patients’ general symptom experience in both frequency and severity, though a variety of symptoms were still experienced at least occasionally by most participants. The most common symptoms still experienced are abdominal pain (*n* = 8, 100% adults; *n* = 34, 97% children), diarrhea (*n* = 7, 88% adults; *n* = 32, 91% children), excessive gas (*n* = 6, 75% adults; *n* = 23, 66% children), bloating (*n* = 6, 75% adults; *n* = 20, 57% children), and reflux (*n* = 5, 63% adults; *n* = 18, 51% children). See Table 2 for the full list of current symptoms. Below are sample quotes describing how the symptom experience improved post-sacrosidase treatment.

- *…it’s not as bad as it used to be…but I do have pain and…nausea. (Pediatric participant)*
- *…having a lot of stomach pain was the most common symptom that I had and still have every now and then. (Adult)*
- *…we still have the occasional diarrhea, but it’s not quite as bad. (Caregiver)*
- *Yes, [gas] used to be like a lot more frequent but now it’s like a lot less. (Pediatric participant)*

**Table 2:** Current symptoms

Symptom	Adults (*n* = 8)		Caregivers/children (*n* = 35)		Total (*N* = 43)	
	*n*	%	*n*	%	*n*	%
Abdominal pain	8	100	34	97	42	98
Diarrhea	7	88	32	91	39	91
Excessive gas	6	75	23	66	29	67
Bloating	6	75	20	57	26	60
Reflux	5	63	18	51	23	53
Loss of appetite	2	25	17	49	19	44
Nausea	5	63	14	40	19	44
Excessive daytime hunger	1	13	17	49	18	42
Constipation	3	38	12	34	15	35
Excessive burping	2	25	13	37	15	35
Vomiting	1	13	14	40	15	35
Floating stools	2	25	12	34	14	33
Change in stool color	2	25	13	37	15	35
Fecal incontinence	0	0	10	29	10	23
Excessive nighttime hunger	1	13	8	23	9	21
Fatigue	2	25	6	17	8	19
Urinary incontinence	1	13	7	20	8	19
Rash	1	13	8	23	9	21
Excessive urination	1	13	5	14	6	14
Headaches	2	25	3	9	5	12
Frequent infections	0	0	5	14	5	12
Feeling hot/sweaty	1	13	3	9	4	9
Bloody stools	1	13	2	6	3	7
Black/tarry stools	0	0	3	9	3	7
Odd smelling stool	0	0	3	9	3	7
Leg pain	0	0	2	6	2	5
Stomach bubbling	1	13	1	3	2	5
Lack of weight gain	0	0	1	3	1	2
Dehydration	0	0	1	3	1	2
Dizziness	0	0	1	2	1	2
Hyperactive	0	0	0	0	0	0
Night terrors	0	0	1	3	1	2
Pale skin	0	0	1	3	1	2
Shiners around eyes	0	0	1	3	1	2
Throat clearing	0	0	1	3	1	2
Sleep difficulties	0	0	0	0	0	0

Most participants reported administering sacrosidase as prescribed, mixing it either with milk or water prior to administration, and taking it with every snack and meal. A little over half (*n* = 24, 56) of participants reported that they never alter their dose, while 44% did, at least occasionally, alter the dose. Reasons for increasing the dose included when planning to eat sweets, an increase in symptom frequency, weight changes, or when eating larger portions. When asked what they liked about sacrosidase, participants most often noted its effectiveness, specifically related to their ability to eat a wider variety of foods—including sweets—and the improvement in CSID symptoms. Participants noted the need to keep sacrosidase refrigerated and the high frequency of administration as common dislikes. After receiving the confirmed CSID diagnosis, participants generally reported making diet changes as part of their treatment plan. The changes were generally focused on increased restrictions on their sucrose and starch intake as it is these types of foods that could trigger CSID symptoms, such as abdominal pain, diarrhea, and gas. While most participants felt sacrosidase was effective in allowing a healthier diet with a wider variety of foods to choose from, many still maintained some limitations on starch and sucrose intake, particularly starch. Participants talked about the ability to eat foods with sacrosidase that they couldn’t eat before as helping them to feel more “normal,” as indicated by the quotes below.

- *That is pretty much my favorite part of [sacrosidase]. It allowed him to eat things and it’s…it’s helped us more to be able to raise him as a normal […] 3-year-old who can try more foods and have that experience. (Caregiver)*
- *…I can tell because I am definitely not as sick and like I started immediately getting healthier once I like was first put on the medicine, and I was always sick when I was little, and I like could not control any of it and once I started taking [sacrosidase] it like immediately started helping me with like the stomach pain… (Pediatric participant)*
- *…I really like that it works for me, that I’m able to eat most foods…especially sweets and have…less symptoms than I would without it…I know that a lot of people with my condition …for them it works better to just have a sucrose-free diet and I’m really thankful that I don’t have to do that and that I am able to still feel somewhat normal and eat normally. (Adult)*

### Impacts on daily life

Even with treatment, participants reported experiencing a variety of daily life impacts related to CSID. Most participants (n = 38, 88%) reported that one or more daily activities were impacted due to CSID. Many patients/caregivers reported having to stop doing an activity or avoiding it all together. Some examples of these activities, particularly for the pediatric sample, include, playing outside, sports, and visiting friends. Abdominal pain and diarrhea were the two symptoms most commonly reported as being the cause of disruption in daily life.

- *Uh, yeah. If I’m outside or something, I…and it…uh, I guess it just sort of hits me, and I just have to stop what I’m doing and go use the bathroom. (Pediatric participant)*

Seventeen (40%) participants (*n* = 4, 50% adults; *n* = 13, 37% children) reported missing partial or full days of school because of CSID. The most cited reasons for missing school included pain diarrhea, and vomiting. Other school impacts included decreased focus and limits on participation in school activities, such as snack time, school parties, and class field trips.

- *…if I have a really bad pain, I might not even leave the house that day. I might not be able to go to school. (Adult)*
- *[…]there would be times I would need to pick her up from school because she wanted to come home and have diarrhea, … or she just wasn’t feeling well and didn’t feel like she can stay the rest […] of the day. […] I would say each year she misses about 25 days a year […] So I…I would say it is impactful to school. (Caregiver)*

Some adult participants reported experiencing work impacts. Two (25%) adults reported missing full or partial days of work due to CSID, and two (25%) adults reported that prior to treatment with sacrosidase they were unable to work outside of the home.

### Social and relationship impacts

Many participants noted that, even with treatment, living with CSID impacted their social lives, both in their ability to participate in social activities and in their relationships. Diet limitations were particularly impactful, with participants most frequently reporting the limitations experienced on eating out or attending social events. Patients and caregivers reported having to make accommodations when attending events, such as bringing their own food or eating beforehand, and sometimes choosing not to attend at all. Participants also noted that it was difficult to find restaurants to accommodate their diet. Some reported that in social situations, the diet limitations or the need to take medication prior to eating, made them feel different or embarrassed, and a few noted being excluded from social events due to their diet limitations.

- *I know she doesn’t get invited sometimes because people don’t want to have to … worry about the diet even though we’ve always made it about not worrying about the diet… (Caregiver)*
- *Well, it’s like…ah, it’s kind of like, uh, setting me back a little ‘cause like I like hanging out with friends a lot, and it’s like we’ll always go out to eat somewhere, and then I’m the like awkward one that has to like bring out my medicine, and it takes like a minute or two to like do it, and everyone’s like watching you, and then it’s just like, whatever, like I do have symptoms and like I’ll have to go to the bathroom. It’s like embarrassing because I don’t know how long I’ll be in there, uh, going. (Pediatric)*

Participants also reported CSID-related relationship impacts in all areas. Impacts on family relationships included both close and extended family members. The impacts were often related to diet, with some caregivers reporting that family members were not respectful or supportive of the patient’s diet limitations, while others noted frustration with restrictions on buying and preparing food within the home.

- *Because it costs more…you know, like sometimes it costs more ‘cause I need special foods and things and my husband gets kind of stressed about how we need so much special things. (Adult)*
- *…sometimes the whole family inner dynamics can be hard when you always feel like you’re a burden to social gathering because they have to adapt themselves around you and they all wanna have their potatoes or whatever, but they sort of feel guilty that some of you can’t have potatoes… (Caregiver)*

Several patients/caregivers also noted negative impacts on relationships with friends and classmates due to CSID. Patients may choose not to go over to a friend’s home, especially avoiding spending the night. Feeling socially accepted and avoiding unwanted attention was important to patients.

- *He has friends at school. He has friends at church. Beyond that,… if there’s…like hanging out with friends, um, it’s done in our home. Friends don’t…there’s a few friends that we trust that will take him for like a day or something and they’ll, um, and they do well with him but that’s a very…very rare event that he actually goes to somebody’s house without mom or dad present. (Caregiver)*
- *He has, you know,…just a handful of friends that he will go stay with. And spend the night because he doesn’t want everybody to know. I mean all the kids at the school know he takes his medicine obviously at lunch, but he doesn’t wanna get into detail with a lot of them, so just the handful that he’s been raised with.(Caregiver)*

### Emotional impacts

A majority of patients and caregivers reported emotional impacts related to having CSID. The three most commonly reported emotional impacts were irritability, embarrassment, and an awareness or feeling of being different or left out. Other commonly reported emotional impacts included frustration, annoyance, and anxiety.

Specific signs and symptoms were often linked to particular emotional impacts. For example, irritability or “grumpiness” was often linked to abdominal pain, while embarrassment was associated with diarrhea, frequent bathroom visits, fecal incontinence, or the need to take medication in front of others.

Caregivers/patients described the feeling of being different or left out in relation to diet limitations and an inability to eat food served at social events and the need to take medication in front of others.

- [referring to bloating] *It’s embarrassing. I feel like it’s noticeable. I don’t know if it is but I just…it’s…I feel like…it’s embarrassing because I feel skinny in the morning and then be so uncomfortable and I feel like it’s noticeable that I’m bigger. (Adult)*
- *He’s different and he doesn’t like being different. He wants to be just like everybody else sometimes. (Caregiver)*
- *Um, after I was diagnosed, my mom put me on a super restrictive diet that just…it wasn’t bad but when I saw people like eating candy that I couldn’t have, that’s when it was bad. ‘Cause then I kind of felt like left out and stuff. (Pediatric participant)*
- *Um, he’s very irritable. He’s very grouchy. (Caregiver)*

## Discussion

This is the first qualitative study in CSID aimed at assessing the patient and caregiver journey from symptom onset to diagnosis and after starting treatment. Patient and caregiver insight are essential for improving the understanding and awareness of this disorder by both HCPs and the wider community, as well as to help fill the gaps in natural history data that are so often present in rare disease research [29].

Encouragingly, there is a growing emphasis on patient-centered research [27, 33]. Researchers investigating GI diseases such as IBS [34] inflammatory bowel disease (IBD) [35–40], and celiac disease [41] are increasingly considering the patient’s perspective and encouraging partnerships between HCPs and patients. Such research, especially in rare diseases like CSID, is vital to ensure the outcomes most important to patients are identified and result in a patient-centered approach to management of CSID and other similar disorders.

The findings from this study highlight the need for greater awareness regarding CSID. The diagnosis itself is not difficult to make, but the journey to receive an accurate diagnosis can be long and problematic, as reported by the patients and caregivers in this study. Because symptoms of CSID (e.g., diarrhea, gas, abdominal pain, and bloating) mimic other more common GI disorders, patients often saw many different HCPs and underwent a wide variety of more routine diagnostic tests for other diseases and disorders before receiving an accurate diagnosis of CSID. Half of the patients (22, 51%) were diagnosed with CSID before three years of age, and all but four patients were diagnosed before the age of 15. However, for over a quarter of patients, the diagnostic process took three or more years.

Patients and caregivers reported that sacrosidase played an important role, along with a low-starch and low-sucrose diet, in the successful management of CSID, enabling patients to eat a wider variety of foods. Sacrosidase helped patients reduce the severity and/or frequency of symptoms when taken as prescribed. However, patients still experienced a variety of symptoms, most commonly occasional diarrhea, gas, and abdominal pain, which typically occurred after having eaten too much sucrose or starch.

Patients and caregivers reported experiencing a variety of impacts related to CSID, including social, emotional, and work/school-related impacts. While treatment with sacrosidase improved patients’ health-related quality of life through a reduction in symptoms and the ability to eat a wider variety of foods, impacts were not eliminated, and some patients reported additional impact (e.g., embarrassment) related to taking the medication itself. Due to the frequency with which social and emotional impacts were reported, this research indicates that patients and caregivers may benefit from targeted emotional and psychological support (e.g., individual and group counseling, support groups) in addition to dietary support.

Education is key for improving outcomes for those diagnosed with CSID. Educating the medical community about identifying, diagnosing, and treating CSID would help reduce the time it takes for patients to reach a CSID diagnosis. Patients and caregivers need access to dietitians who have experience with CSID and can provide individualized nutrition counseling to reduce the lingering GI symptoms that have been reported in this study and improve patient outcomes. Credible educational resources and patient support groups would also benefit patients with CSID, and increasing public awareness of CSID could hopefully reduce the social stigma experienced by many patients living with CSID.

This study had several limitations. There is a potential for selection bias as participants taking part in the interview portion of the study were limited to those participating in a larger observational study. There may also be recall bias, as caregivers and patients were asked to talk about their whole journey, which may have been over a period of many years. The small adult sample size limits the generalization of outcomes from this survey to all adults with CSID. The outcomes from this study conducted in the US may not be generalizable to CSID patients from different countries, where different medical practices and cultural food and diet norms may exist.

## Conclusion

CSID is a disorder that affects patients’ health-related quality of life. A lack of knowledge in the health care community about CSID can be a barrier to diagnosis. Therefore, educating HCPs about CSID may be helpful in decreasing what can be a long and burdensome diagnostic process for both patient and caregiver. Patients and caregivers reported being able to successfully manage CSID with sacrosidase and a low-starch and low-sucrose diet. Sacrosidase reduced the severity and frequency of GI symptoms and allowed patients to consume a wider variety of foods that they would otherwise be unable to eat. However, even with treatment, participants still experienced a variety of impacts on their daily life related to having CSID, indicating areas of potential unmet needs. As this research is the first of its kind in this population, additional research, both qualitative and quantitative, will be important to further broadening the understanding of health-related quality of life impact and unmet need experienced by this population and identifying ways to best meet those needs.

## Data Availability

Interview materials and data are available upon request.

## Code availability

MAXQDA v.11 was used to conduct the qualitative analysis.
